# Supplementary material for: MetaRibo-Seq measures translation in microbiomes
Source: Nat Commun. 2020 Jun 29;11:3268. doi: 10.1038/s41467-020-17081-z (PMC7324362; doi:10.1038/s41467-020-17081-z)
Supplement: Supplementary file 10 — Supplementary Data 7 [file 41467_2020_17081_MOESM10_ESM.zip › File2/Confidence_High_Taxonomy/266254_out.krona.html]

Javascript must be enabled to view this page.

members
magnitude
magnitudeUnassigned
count
unassigned
taxon
rank

266254\_out

4

2
superkingdom
4

1239
4
phylum

909932
4
class

order
4
1843488

909930
family
4

33024
genus
4

33025

SRS022713\_contig\_number\_7566SRS074670\_contig\_number\_8233SRS148196\_contig\_number\_37912SRS148721\_contig\_number\_41183
4
species
